# Preclinical Study of Novel Gene Silencer Pyrrole-Imidazole Polyamide Targeting Human TGF-β1 Promoter for Hypertrophic Scars in a Common Marmoset Primate Model

**DOI:** 10.1371/journal.pone.0125295

**Published:** 2015-05-04

**Authors:** Jun Igarashi, Noboru Fukuda, Takashi Inoue, Shigeki Nakai, Kosuke Saito, Kyoko Fujiwara, Hiroyuki Matsuda, Takahiro Ueno, Yoshiaki Matsumoto, Takayoshi Watanabe, Hiroki Nagase, Toshikazu Bando, Hiroshi Sugiyama, Toshio Itoh, Masayoshi Soma

**Affiliations:** 1 Department of General Medicine, Nihon University School of Medicine, Tokyo, Japan; 2 Division of Life Science, Advanced Research Institute for the Sciences and Humanities, Nihon University Graduate School, Tokyo, Japan; 3 Division of Nephrology Hypertension and Endocrinology, Department of Medicine, Nihon University School of Medicine, Tokyo, Japan; 4 Marmoset Research Department, Central Institute for Experimental Animals, Kanagawa, Japan; 5 Department of Clinical Pharmacokinetics, College of Pharmacy, Nihon University, Chiba, Japan; 6 Department of Cancer Genetics, Chiba Cancer Center Research Institute, Chiba, Japan; 7 Department of Chemistry, Kyoto University Graduate School, Kyoto, Japan; University of Texas Health Science Center, UNITED STATES

## Abstract

We report a preclinical study of a pyrrole-imidazole (PI) polyamide that targets the human transforming growth factor (hTGF)-β1 gene as a novel transcriptional gene silencer in a common marmoset primate model. We designed and then synthesized PI polyamides to target the hTGF-β1 promoter. We examined effects of seven PI polyamides (GB1101-1107) on the expression of hTGF-β1 mRNA stimulated with phorbol 12-myristate 13-acetate (PMA) in human vascular smooth muscle cells. GB1101, GB1105 and GB1106 significantly inhibited hTGF-β1 mRNA expression. We examined GB1101 as a PI polyamide to hTGF-β1 for hypertrophic scars in marmosets *in vivo*. Injection of GB1101 completely inhibited hypertrophic scar formation at 35 days post-incision and inhibited cellular infiltration, TGF-β1 and vimentin staining, and epidermal thickness. Mismatch polyamide did not affect hypertrophic scarring or histological changes. Epidermis was significantly thinner with GB1101 than with water and mismatch PI polyamides. We developed the PI polyamides for practical ointment medicines for the treatment of hypertrophic scars. FITC-labeled GB1101 with solbase most efficiently distributed in the nuclei of epidermal keratinocytes, completely suppressed hypertropic scarring at 42 days after incision, and considerably inhibited epidermal thickness and vimentin-positive fibroblasts. PI polyamides targeting hTGF-β1 promoter with solbase ointment will be practical medicines for treating hypertrophic scars after surgical operations and skin burns.

## Introduction

Novel translational medicines such as molecularly-targeted drugs and monoclonal antibodies have been introduced into clinical practice [1]. In spite of the discovery of these agents, many diseases cannot be treated with the present medicines. Thus, nucleic acid medicines like antisense deoxynucleotides and ribozymes and decoys were developed as next-generation therapeutic medicines. However, these agents can be easily degraded by nucleases like DNase or RNase, and thus efficient systems of drug delivery are required for adequate distribution in organs.

Pyrrole-imidazole (PI) polyamides are novel gene silencers that were initially identified from antibiotics such as distamycin A and duocarmycin A. The small synthetic molecules of PI polyamides consist of aromatic rings of amino acids N-methylpyrrole and N-methylimidazole that recognize and bind to DNA with sequence specificity [2,3]. Due to their ability to bind with high affinity and specificity to double-helical DNA, PI polyamides can inhibit protein interaction, including DNA transcription factor [4,5]. Recognition of DNA depends upon the pairing of Pyrrole (Py) and Imidazole (Im) side by side in the minor groove. Pairing of Im opposite Py will target the G-C base pair, whereas the Py-Im pairing will target the C-G base pair. The Py-Py pairing targets A-T and T-A base pairs degenerately [3]. PI polyamides are fully resistant to the biological degradation that is induced by nucleases. PI polyamides do not require vector-assisted delivery systems because of their cell permeability, and they may easily enter nuclei. Thus, the applicability of PI polyamides as novel gene therapy agents might be greater than that of nucleic acid medicines [6]. Various sequence-specific DNA-binding PI polyamides have been created to control gene expression. We previously reported that PI polyamides targeting human TGF-β1 (hTGF-β1) promoter significantly inhibit both TGF-β1 promoter activity and the expression of TGF-β1 mRNA and protein in human cells [7,8]. Thus, synthetic PI polyamides that can target gene promoters might be potentially effective as practical medicines for regulating gene transcription. We demonstrated that intravenously injected fluorescein isothiocyanate (FITC)-labeled PI polyamide localized strongly to nuclei in the kidney, aorta, lung and liver without any drug delivery system [8]. PI polyamides bind to the chromosome in a sequence specific manner. Intravenously injected PI polyamides remain in the nuclei of the kidney for more than one week [9]. We have demonstrated that PI polyamide targeting TGF-β1 effectively attenuates progressive renal diseases [9]; stenosis of the carotid artery after angioplasty [10]; and alkali burn of the cornea [11] in rats. We also demonstrated that PI polyamides to rat TGF-β1 completely suppressed hypertrophic scars [12]. PI polyamides targeting TGF-β1 may thus be a feasible gene silencer for treating hypertrophic scars and keloids.

The current study is a preclinical study to develop PI polyamides targeting the hTGF-β1 gene promoter as a novel transcriptional regulator for practical medicines. We employed a primate model using *Callithrix jacchus* (the common marmoset), which possesses similar genomic structures to humans. We molecularly designed and synthesized PI polyamides targeting hTGF-β1 promoter, performed lead optimization, and examined application of PI polyamides to hTGF-β1 in ointment medicines to treat hypertrophic scarring in marmosets.

## Materials and Methods

### Design and synthesis of PI polyamides to hTGF-β1 promoter

**Fig 1:**
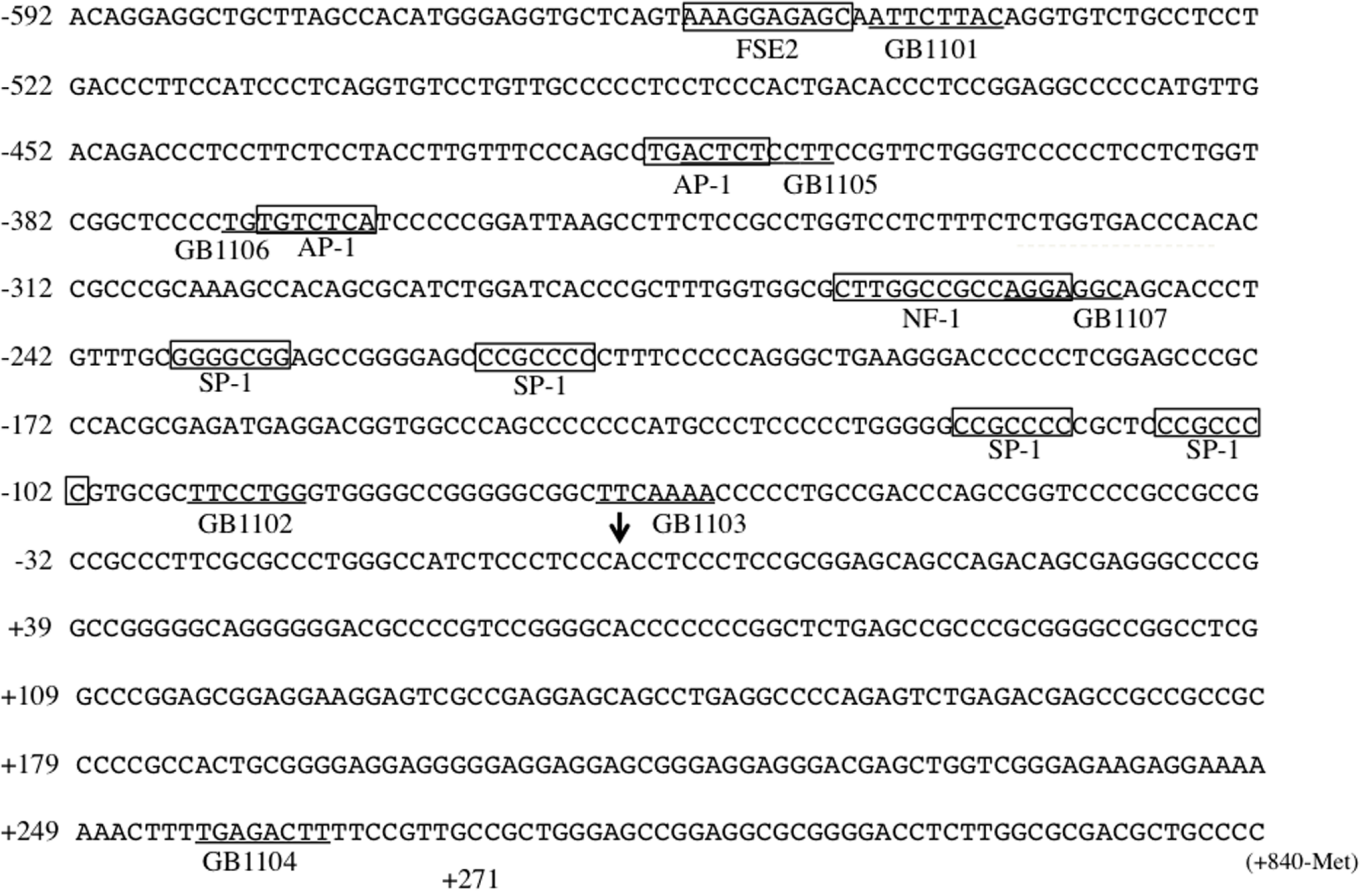
Binding sites of synthetic pyrrole-imidazole (PI) polyamides (GB1101-1107) on human transforming growth factor-β1 promoter sequence. Arrow: Major transcription initiation points, Open box: transcription factor binding sites, Underlines: PI polyamide binding sites. FSE2: fat-specific element 2, AP-1: activating protein-1, NF-1: nuclear factor-1.

**Fig 2:**
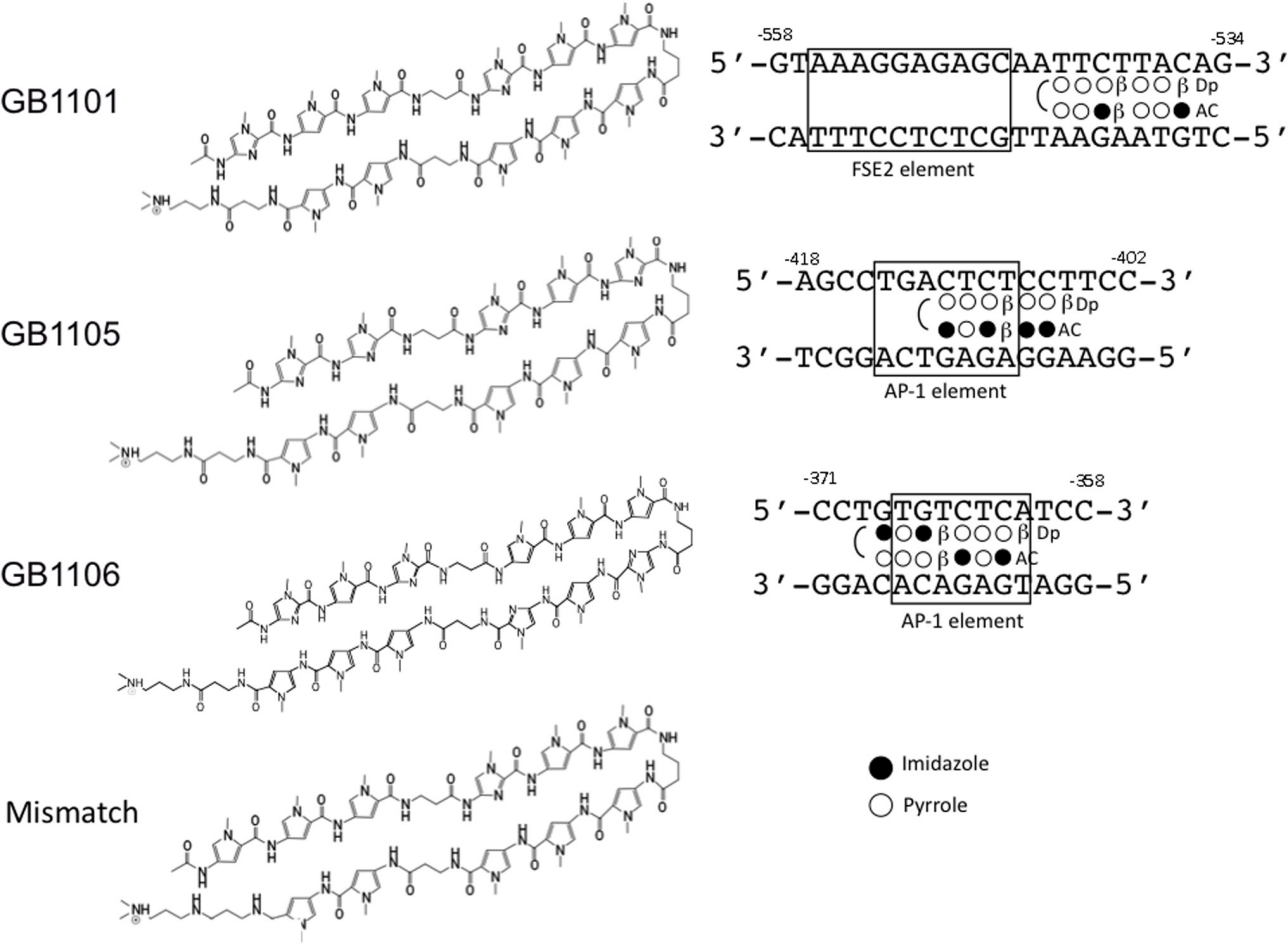
Structures and target sequences of pyrrole-imidazole polyamides (GB1101, GB1105 and GB1106) targeting to the human transforming growth factor-β1 promoter and structure of mismatch polyamide. Box indicates fat-specific element 2 (FSE2) binding element or activating protein-1 (AP-1) binding element.

We designed seven PI polyamides (GB1101-1107) to bind bp -558 to +261 on the hTGF-β1 promoter sequences [13]. GB1101 is adjacent to the adipocyte P2 gene contains a regulatory element (FSE2) binding site. GB1102, GB1103 and GB1104 are adjacent to promoter sequences that are between humans and rats. GB1105 and GB1106 are adjacent to two AP-1 binding sites. GB1107 is adjacent to the NF-1 binding site (Fig 1). Fig 2 shows the structures of GB1101, GB1105 and GB1106 and their binding sites. We designed GB1105 and GB1106 to span the boundary of the AP-1 binding site (box) of the hTGF-β1 promoter sequence.

We induced Im and Py substitution to create the PI polyamides. Machine-assisted automatic synthesis of hairpin-type PI polyamides was carried out with the use of a continuous-flow peptide synthesizer (PSSM-8, Shimadzu, Kyoto, Japan) at 0.1 mmol scale (200 mg of Fmoc-b-alanine-CLEAR Acid Resin, 0.50 meq/g, Peptide Institute, Osaka, Japan). Automatic solid phase synthesis was performed by washing with dimethylformamide (DMF); removing the Fmoc group using 20% piperidine/DMF; washing with methanol; coupling with a monomer for 60 min in an environment of 1-[bis(dimethylamino)methylene]-5-chloro-1H-benzotriazolium 3-oxide hexafluorophosphate (HCTU) and diisopropylethylamine (4 eq each); washing with methanol; protecting with acetic anhydride/pyridine; and washing with DMF as the final step. After the Fmoc group was removed from the Fmoc-β-alanine-Wang resin, successive of washes of the resin with methanol were performed. The coupling step was carried out with Fmoc-amino acid and followed by a methanol wash. We repeated these steps until the entire sequencing was complete. After completion of the coupling steps, the N-terminal amino group was protected and washed with DMF, followed by draining of the reaction vessel. We next isolated the synthetic polyamides after the cleavage step (5 ml 91% trifluoroacetic acid-3% triisopropylsilane-3% 5 dimethylsulfide-3% water/0.1 mmol resin) by cold ethyl ether precipitation. The synthetic polyamides were isolated after the cleavage step (5 ml N, N-dimethylaminopropylamine/0.1 mmol resin, 50°C overnight) by cold ethyl ether precipitation. To purify the polyamides, high-performance liquid chromatography (HPLC) was performed with use of a PU-980 HPLC pump, UV-975 HPLC UV/VIS detector (Jasco, Easton, MD), and Chemcobound 5-ODS-H column (Chemco Scientific, Osaka, Japan).

### Cell culture

Human vascular smooth muscle cells (VSMCs) (Cambrex Bio Science Rockland, Inc., Rockland, ME) were maintained in Dulbecco’s modified Eagle’s medium (DMEM) supplemented with 10% fetal calf serum (Invitrogen, Carlsbad, CA) and 50 mg/ml streptomycin (Invitrogen). After it reached confluence (7–10 days after seeding 1 vial of 10^5^ cells/cm^2^), the VSMC culture displayed a typical hill-and-valley pattern. Marmoset fibroblasts were prepared from newborn skin by digestion with 5 mg/ml collagenase type I (Sigma, St. Louis, MO) overnight. They were cultured in DMEM supplemented with 10% fetal calf serum, 0.1 mg/ml penicillin and 0.05 mg/ml gentamicin. Cells were passaged by trypsinization with 0.05% trypsin (Gibco Life Technologies, Gaithersburg, MD) and plated in 6- or 24-well culture dishes at a density of 10^5^ cells/cm^2^. Cells were cultured in a water-saturated CO_2_ incubator at 37°C.

### RNA extraction and real-time PCR analysis

TRIzol reagent (Invitrogen, CA) was used to extract total RNA from cultured cells. The total RNA (1 μg) was then reverse transcribed into cDNA with random 9-mers with a Takara RNA PCR Kit (AMV) Ver. 3.0 (Takara Bio, Ohtsu, Japan). Assay-on-Demand primers and probes (human TGF-β1: Hs00998133_m1) were obtained from Applied Biosystems Life Technologies (Tokyo, Japan). An ABI Prism 7300 (Applied Biosystems) was used to quantify mRNA. Each sample (each reaction, 5 μl complementary DNA; total volume, 25 μl) was run in triplicate. To control sample loading, we determined 18S ribosomal RNA levels with TaqMan Ribosomal RNA Control Reagents (Applied Biosystems). Amplification conditions included 50°C for 2 min, 95°C for 10 min, 60 cycles of denaturation (95°C for 15 sec) and combined annealing-extension (60°C for 1 min). We determined the threshold cycle (Ct) and calculated relative quantification of marker gene mRNA expression with the comparative Ct method.

### Ethics

This study conformed to the standards of the US National Institute of Health’s *Guide for the Care and Use of Laboratory Animals* (NIH Publication No. 85–23, revised 1996). The Nihon University IACUC committee approved this study, which was conducted in accordance with the Guidelines for Conducting Animal Experiments of the CIEA.

### Animals

We used total nine male common marmosets (Callithrix jacchus) obtained from CLEA Japan, Inc. (Tokyo, Japan) used in this study. Experiments were performed in the Central Institute for Experimental Animals (CIEA). The marmosets were housed in pairs in stainless steel living cages (39655670 cm) with 45–55% humidity and illumination for 12 hours per day. Wood perches for locomotion and a platform for a bed were placed in each cage for environmental enrichment. Marmosets were kept healthy and nourished with a balanced diet (CMS-1M; CLEA Japan Inc.), including mixed L(+)-ascorbic acid (Nacalai Tesque, Tokyo, Japan), vitamins A, D3, and E (Duphasol AE3D; Kyoritsu Seiyaku Co., Ltd., Tokyo, Japan), and honey (Nihonhatimitsu Co., Ltd., Gifu, Japan). The diet moistened with hot water was fed in the morning and the dry diet was fed in the afternoon. In addition to the normal diets, sponge cakes, biscuits or apple jelly were fed when humans contacted the marmosets. In each cages there were puzzle feeders. The animals were supplied with tap water *ad libitum* from feed valves. All marmosets were not sacrificed for experiments of the creating skin hypertrophic scar.

### Creation of hypertrophic scars in marmosets

Hypertrophic scars were created on the abdomen skin of the marmoset by scalpel incision. Adult male marmosets weighing 300–350 g were anesthetized by ketamine and isoflurane for inhalation anesthesia. Antibiotic (ampicillin) was injected intramuscularly in the wound just after skin incision. Fluid replacement was injected subcutaneously in the wound after skin incision to prevent dehydration. Four full-thickness linear incisions of 2 cm in length were made down to and including the panniculus carnosus on the abdominal skin of marmosets. The incisions were made equidistant from the midline and adjacent to the four limbs. Two incisions were made in 1 marmoset. One hundred micrograms of GB1101 and mismatch PI polyamides dissolved in 500 μl of H_2_O were injected subcutaneously in the wound site just before the skin incisions.

### Histopathological and immunohistochemical examination

Skin samples obtained from scar lesions and control sites were fixed in 10% neutral buffered formalin and embedded in paraffin. Sections of 6 μm in thickness were then stained with hematoxylin-eosin, deparaffinized, dehydrated in a routine manner and incubated overnight at 4°C with TGF-β1 anti-human TGF-β1 rabbit polyclonal antibody (Yanaihara, Shizuoka, Japan) (1:1000) and anti-human vimentin mouse monoclonal antibody (Novocastra, Tokyo, Japan) (1:1000). The vimentin-positive area was calculated from a sum of the representative vimentin-stained areas using Photoshop CS3 extended (Adobe Systems Inc., San Jose, CA). Four incisions were used for an experiment (biopsy site = 4).

### Distribution of FITC-labeled PI polyamide in skin

To evaluate the most effective ointment for enhancing the distribution of PI polyamides, 10 μg of FITC-labeled GB1101 in 500 μl of 0.02% dimethyl sulfoxide solution mixed with five different ointment bases (Vaseline, a hydrocarbon gel ointment base: Plastibase [Bristol Myers Squibb, New York, NY], hydrophilic ointment [Geritrex Corp., Mt. Vernon, NY], solbase, and Hydroxypropylmethylcellulose [Ashland Inc., Covington, KY]) was subcutaneously injected into skin incisions in rats. In addition, to evaluate the distribution of GB1101 with ointment on skin incisions in marmosets, 10 μg of FITC-labeled GB1101 in 500 μl of 0.02% dimethyl sulfoxide solution mixed with solbase ointment base was applied inside the skin incision. The skin excised at 24 h and 48 h post-injection was embedded immediately in optimal cutting temperature compound (Miles, Elkhart, IN) and frozen in liquid nitrogen. The distribution of the FITC-labeled polyamide was confirmed by fluorescence-inverted microscope (Eclipse TE-2000-U, Nikon, Tokyo, Japan).

### Statistical analysis

Values are shown as the mean ± SE. We used the Student *t-*test for unpaired data and two-way ANOVA with the Bonferroni/Dunn procedure as a post test. A value of *p* < 0.05 was considered to indicate statistical significance.

## Results

### Determination of lead compounds of PI polyamides to hTGF-β1

**Fig 3:**
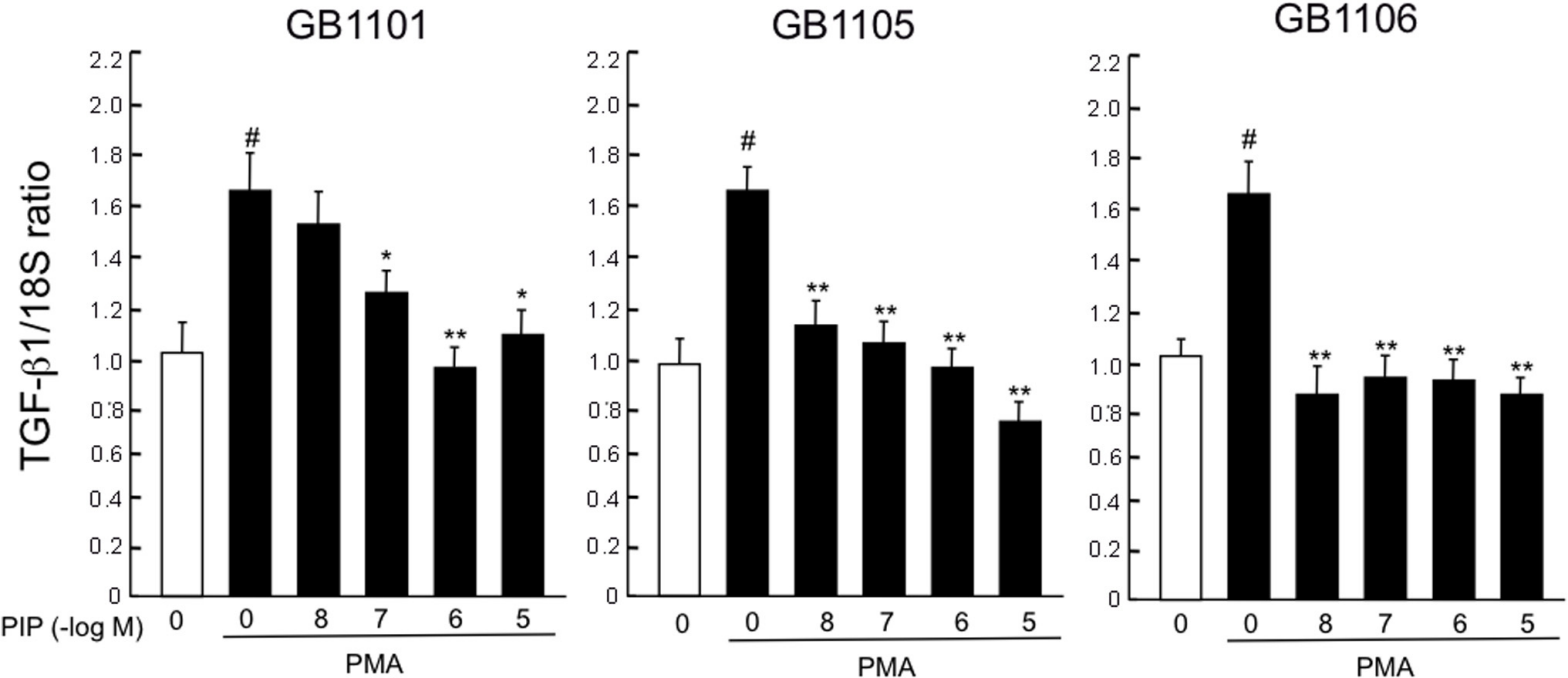
Effects of pyrrole-imidazole (PI) polyamides (GB1101, GB1105 and GB1106) targeting the human transforming growth factor (TGF)-β1 promoter on expression of TGF-β1 mRNA in human vascular smooth muscle cells (VSMCs). VSMCs were incubated with 10^-8^–10^-6^ M PI polyamides targeting human TGF-β1 and mismatch polyamide in the presence or absence of 10^-6^ M phorbol 12-myristate 13-acetate (PMA). Total RNA was extracted, and expression of TGF-β1 mRNAs was evaluated by real-time polymerase chain reaction analysis. Data are mean ± SEM (n = 4). * *p* < 0.05, ** *p* < 0.01 vs. PMA without PI polyamide. # *p* < 0.05 vs. without PMA.

To determine the lead compounds, we examined the effects of seven PI polyamides (GB1101-1107) targeting hTGF-β1 promoter on hTGF-β1 mRNA expression in human VSMCs. Three PI polyamides (GB1101, GB1105 and GB1106) showed significant (*p* < 0.05) inhibition of expression of hTGF-β1 mRNA stimulated with phorbol 12-myristate 13-acetate (PMA). GB1101 and GB1105 significantly inhibited expression of hTGF-β1 mRNA stimulated with PMA in a dose-dependent manner (*p* < 0.05), whereas GB1106 significantly inhibited expression of hTGF-β1 mRNA from 10^-8^ to 10^-5^ M PI polyamides to the same level as baseline expression (*p* < 0.01). Mismatch polyamide did not appreciably inhibit expression of hTGF-β1 mRNA stimulated with PMA (Fig 3). NCBI BLAST Two Sequence Analysis for hTGF-β1 promoter sequences revealed 86% sequence homology between humans and common marmosets, in comparison to 39.5% sequence homology between humans and mice. S1 Fig shows the comparison of human and marmoset TGF-β1 promoter sequence homology by NCBI BLAST Two-Sequence Analysis for GB1101, GB1105 and GB1106. The binding sequences of GB1101 and GB1106 were completely identical. One base in the binding sequence of GB1105 differed between humans and marmosets.

### Effects of PI polyamides to hTGF-β1 on expression of hTGF-β1 mRNA in marmoset fibroblasts

The effects of 10^-9^ and 10^-7^ M GB1101 and GB1106 on expression of TGF-β1 mRNA stimulated with PMA in marmoset-derived fibroblasts is shown in S2 Fig. GB1101 significantly inhibited expression of PMA-stimulated TGF-β1 mRNA, whereas GB1106 did not appreciably inhibit expression of TGF-β1 mRNA in marmoset fibroblasts. Mismatch polyamide did not affect expression of PMA-stimulated TGF-β1 mRNA. We then examined GB1101 as a lead PI polyamide targeting the hTGF-β1 promoter for hypertrophic scars in marmosets *in vivo*.

### Effects of PI polyamides to hTGF-β1 on hypertrophic scars in marmosets

**Fig 4:**
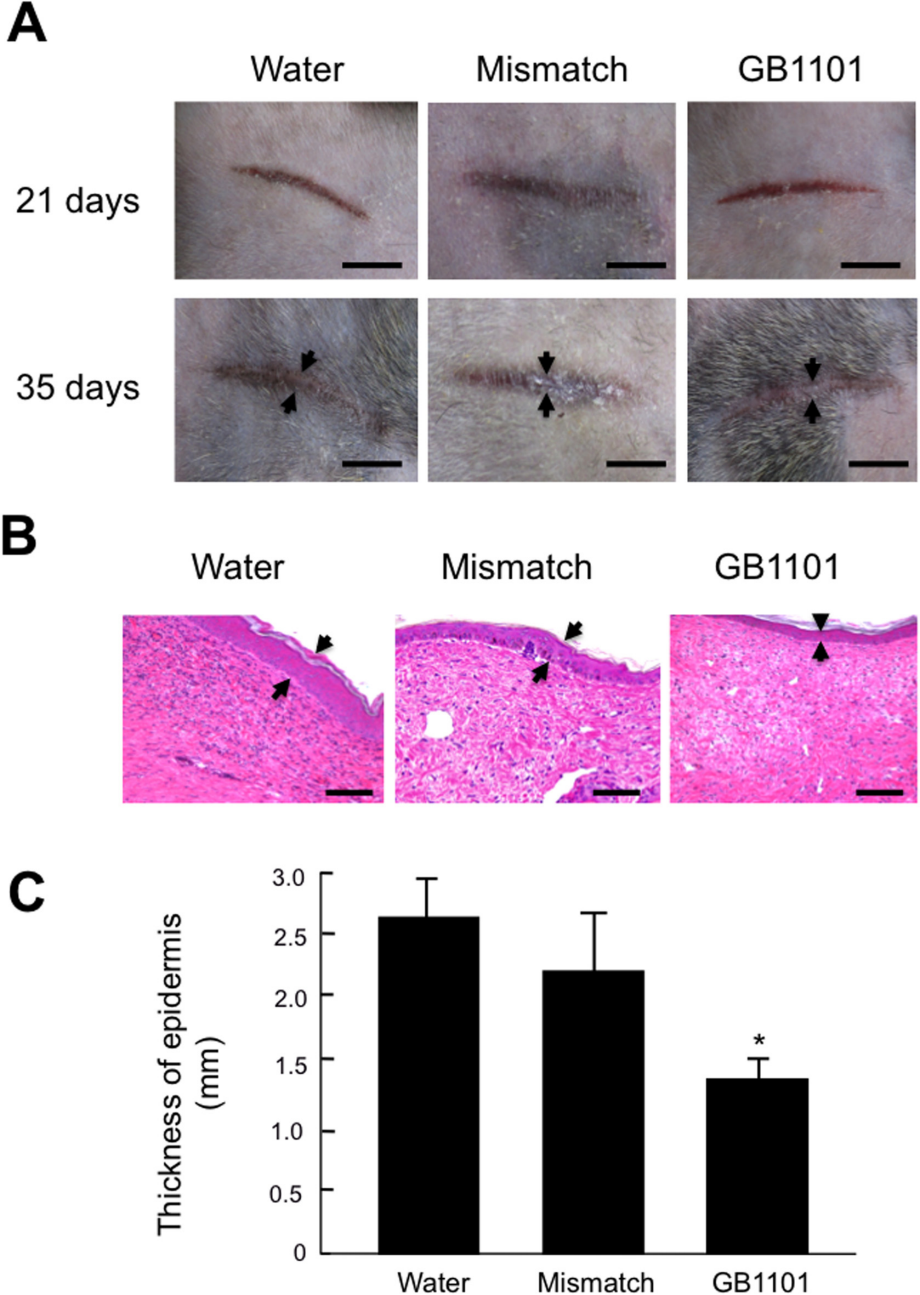
Effects of injection of pyrrole-imidazole (PI) polyamide targeting human transforming growth factor (TGF)-β1 promoter (GB1101) on development of hypertrophic scars. Hypertrophic scars were created on marmoset abdomen skin by scalpel incision. A mixture of 100 μg of GB1101 and mismatch PI polyamides dissolved in 500 μl of H_2_O (Water) was subcutaneously injected into the wound site just before incision. (A) Typical macroscopic changes on post-incision days 21 and days 35 (two experiments) from 5 experiments. Arrows indicate hypertrophic scars. Bar = 5 mm. (B) Sections of 6 μm in thickness at 35 days post-incision from 4 experiments were stained with hematoxylin-eosin. Arrows indicate dermal thickness of hypertrophic scars. Bar = 500 μm. (C) Comparison of water, mismatch PI polyamide and GB1101 injection on epidermal thickness of hypertrophic scars at 35 days post-incision. Values are expressed as mean ± SE (n = 4). * *p* < 0.01 vs. Mismatch PI polyamide.

Macroscopic changes in the incisional wounds in marmosets subcutaneously injected with GB1101 are shown in Fig 4A. One hundred micrograms of GB1101 and mismatch PI polyamides were subcutaneously injected into the wound site just before incision. On days 21 and 35, the incisional wound was elevated and obviously harder to the touch in comparison to the surrounding normal skin. Injection of GB1101 completely inhibited the formation of hypertrophic scars after incision compared to injection of water at 35 days post-incision. Mismatch polyamide did not affect the formation of hypertrophic scars (Fig 4A).

Histological examination of wounds injected with water at 35 days post-incision showed infiltration in the lymphocytes and neutrophils and increases in epidermal thickness. Injection of GB1101 considerably inhibited cellular infiltration and epidermal thickness. Mismatch PI polyamide did not appreciably affect these histological changes (Fig 4B). Epidermal thickness was significantly (*p* < 0.01) lower with GB1101 than with water and mismatch PI polyamide (Fig 4C).

**Fig 5:**
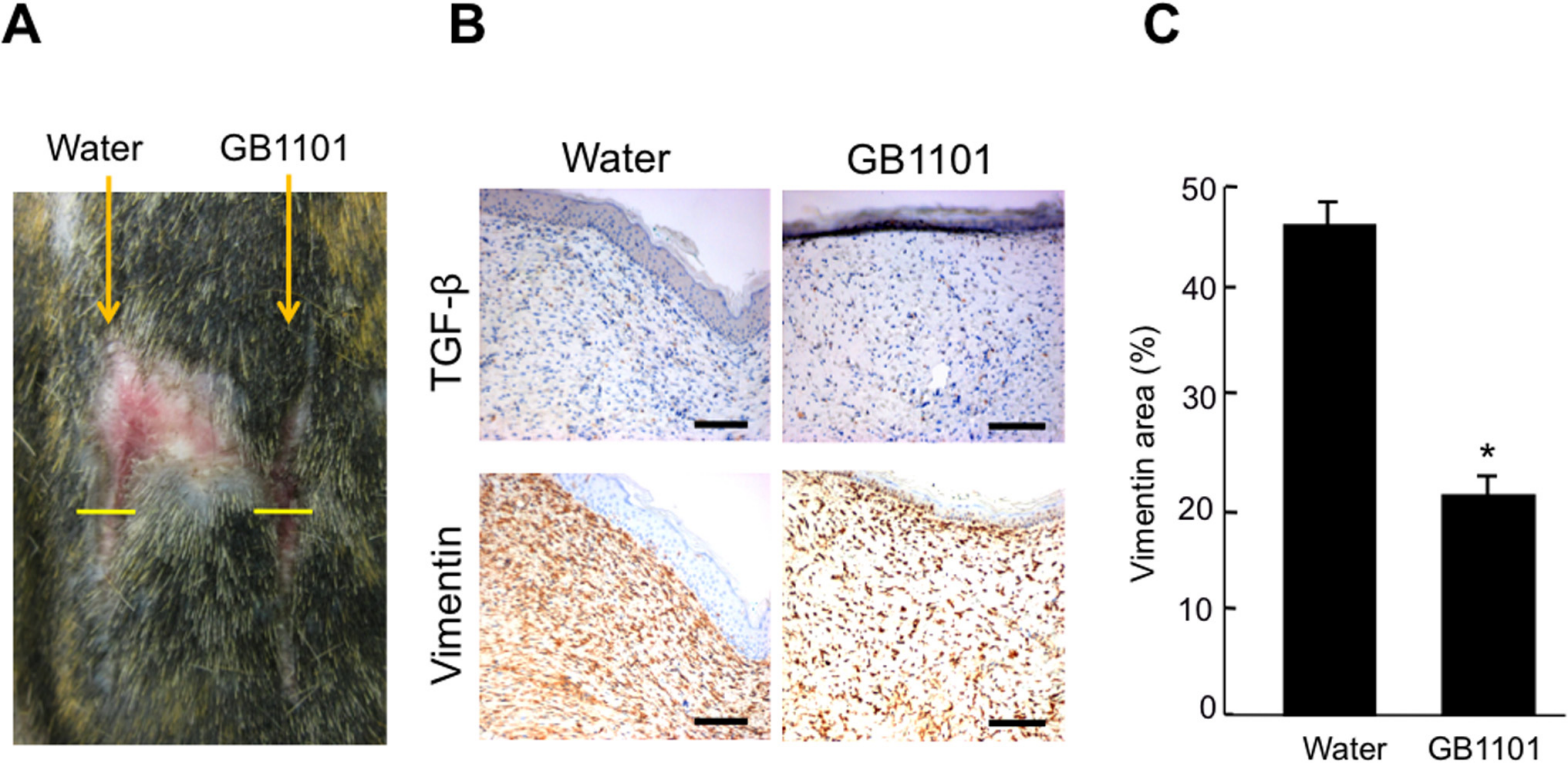
Effects of injection of pyrrole-imidazole (PI) polyamide targeting human transforming growth factor (TGF)-β1 promoter (GB1101) on stainings of TGF-β1 and vimentin at 35 days post-incision. (A) Hypertrophic scars were created on marmoset abdomen skin by scalpel incision. A mixture of 100 μg of GB1101 and mismatch PI polyamides dissolved in 500 μl of H_2_O (Water) was subcutaneously injected into the wound site just before incision. (B) Immunohistochemistry of effects on injection of GB1101 on stainings of TGF-β1 and vimentin in hypertrophic scars at 35 days post-incision. Bar = 500 μm. (C) The vimentin-positive area was calculated from the sum of the representative vimentin-stained areas identified with NIH Image. Data are the mean ±SE (n = 3). * *p* < 0.01 vs. Water.

Immunohistochemistry of the effects of injection of GB1101 on stainings of TGF-β1 and vimentin in hypertrophic scars at 35 days post-incision in marmosets are shown in Fig 5. Injection of GB1101 considerably reduced TGF-β1 staining and the number of vimentin-positive fibroblasts compared with water injection (Fig 5B). The area of vimentin staining was significantly (*p* < 0.01) lower with GB1101 than with water (Fig 5C).

### Distribution of FITC-PI polyamide to hTGF-β1 with ointments into incisions

**Fig 6:**
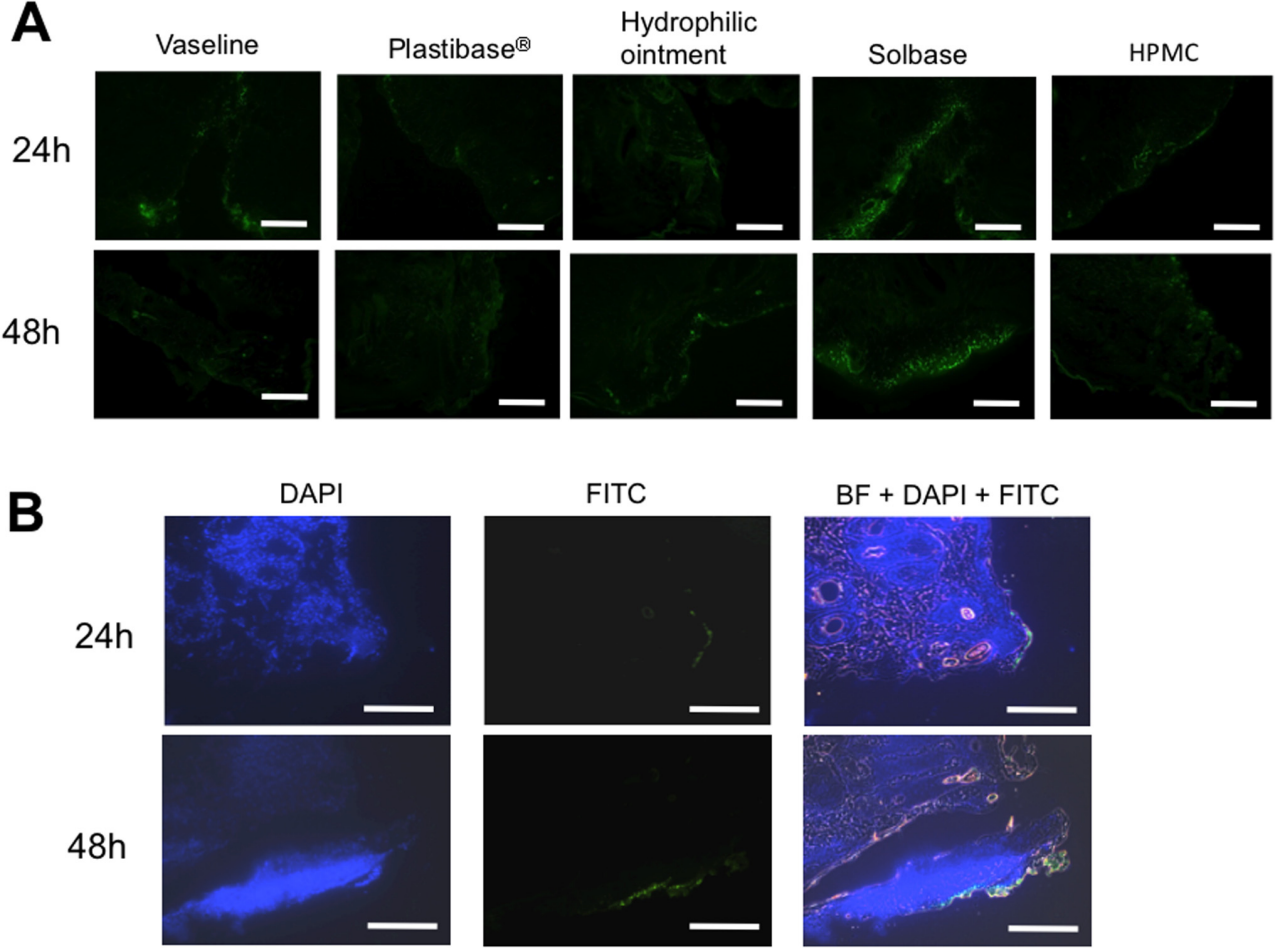
Distribution of FITC-labeled pyrrole-imidazole (PI) polyamide targeting human transforming growth factor (TGF)-β1 promoter in skin incisions. (A) Ten micrograms of FITC-labeled PI polyamide to TGF-β1 GB1101 in 500 μl of a 0.02% dimethyl sulfoxide solution mixed with five different ointment bases (Vaseline, a hydrocarbon gel ointment base: Plastibase, hydrophilic ointment, solbase, and hydrophobized hydroxypropylmethyl cellulose [HPMC]) into skin incisions in rats. (B) Distribution of GB1101 with solbase ointment on skin incision in marmosets. Ten micrograms of FITC-labeled GB1101 with solbase ointment base was applied inside the skin incision. The skin excised at each indicated post-incision time point was embedded immediately in optimal cutting temperature compound and frozen in liquid nitrogen. Nuclei were stained with DAPI. BF: bright field. Bar = 500 μm (original magnification x 400).

Fig 6A shows distribution of FITC-labeled GB1101 with five different ointment bases (Vaseline, a hydrocarbon gel ointment base: Plastibase, hydrophilic ointment, solbase, and hydrophobized hydroxypropylmethyl cellulose) into skin incisions in rats. Among these ointment bases, solbase most efficiently enhanced distribution of FITC-labeled GB1101 in the nuclei of epidermal keratinocytes. We then used solbase as an ointment base for GB1101. Fig 6B shows the distribution of FITC-labeled GB1101 with solbase into skin incisions made in marmosets. FITC-labeled GB1101 was obviously distributed in the epidermis of the incised skin.

### Effects of PI polyamide to hTGF-β1 with solbase ointment on hypertrophic scars in marmosets

**Fig 7:**
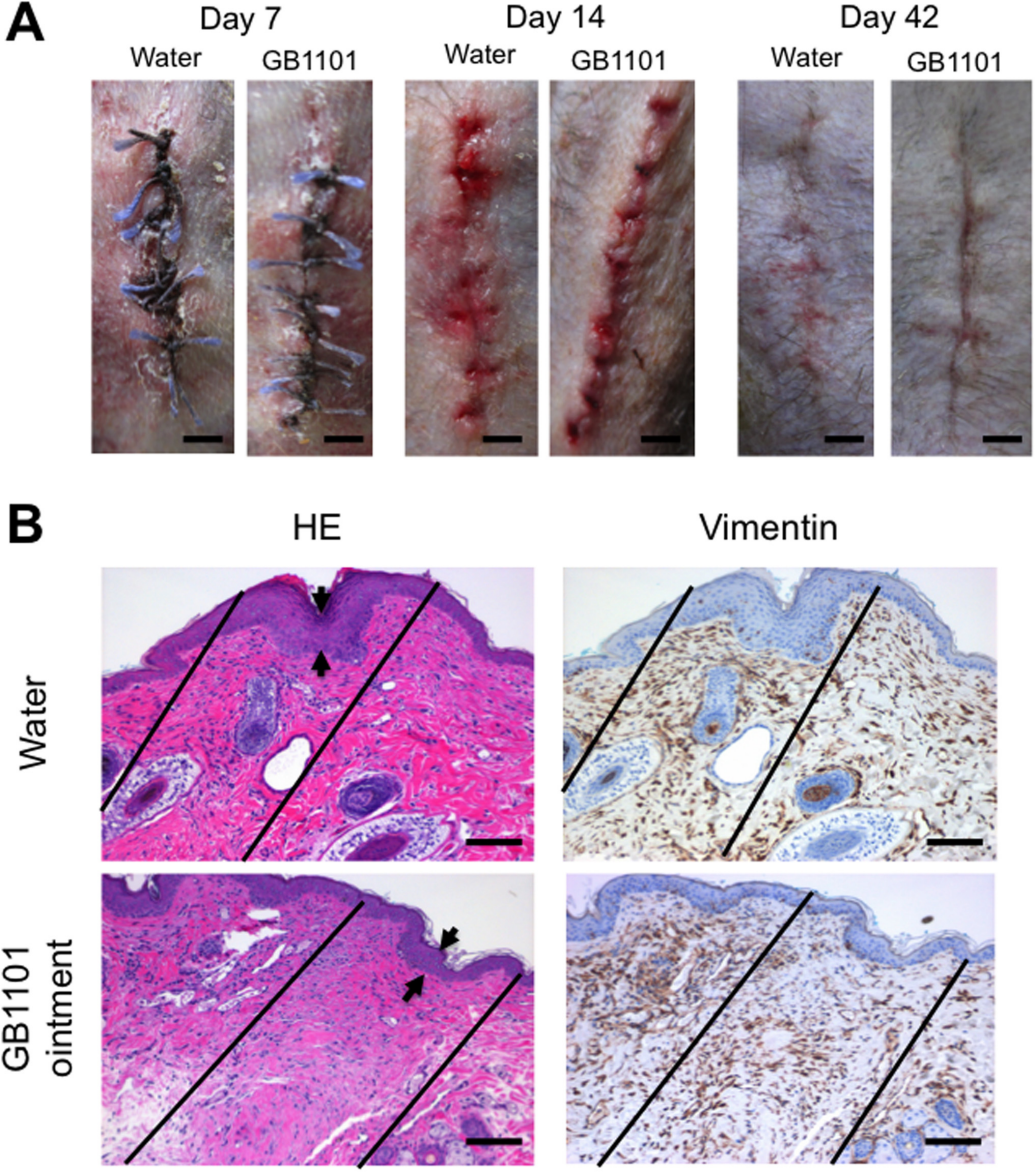
Effects of the application of pyrrole-imidazole (PI) polyamide targeting human transforming growth factor (TGF)-β1 promoter (GB1101) mixed with solbase ointment on development of hypertrophic scars in marmosets. Hypertrophic scars were created on marmoset abdomen skin by scalpel incision. Thirty micrograms of GB1101 with solbase ointment base was applied inside the skin incision. (A) Typical macroscopic changes in the incisional wounds in marmosets subcutaneously injected with PI polyamides on days 7, 14 and 42 from three experiments. Bar = 3 mm. (B) Typical histological evaluation of the effects of PI polyamides on the hypertrophic scars of marmoset skin from three experiments. Sections of 6 μm in thickness were stained with hematoxylin-eosin and used to evaluate vimentin immunohistochemically. Two lines indicate application areas of the ointment. Arrows indicate thickness of dermal thickness hypertrophic scars. Bar = 500 μm.

The effects of the application of GB1101 with solbase ointment on hypertrophic scars caused by incision in marmosets are shown in Fig 7. GB1101 with solbase ointment completely suppressed hypertrophic scarring at 42 days post-incision compared to water with solbase in marmosets (Fig 7A). Histological examination of wounds injected with water at 42 days post-incision showed increases in epidermal thickness and the number of fibroblasts positive for vimentin. Application of GB1101 with solbase ointment considerably inhibited epidermal thickness and the number of fibroblasts positive for vimentin (Fig 7B).

## Discussion

In the development of gene therapies, including nucleic acid medicines, preclinical studies using suitable animal models are important for evaluating safety and therapeutic efficacy. Non-human primate models are required for the preclinical study of medicines that recognize human genes. Because PI polyamides are a novel gene silencer through their transcriptional inhibition of human genes, we used the common marmoset for drug development involving PI polyamides targeting hTGF-β1 promoter. The analysis for TGF-β1 promoter sequences revealed that the homology between humans and marmosets was obviously higher (86%) than that between humans and mice (39.5%). The binding sequences of GB1101 and GB1106 were completely identical between human and marmoset promoter sequences, and GB1101 significantly inhibited expression of PMA-stimulated TGF-β1 mRNA in marmoset fibroblasts whereas GB1106 did not. GB1101 was thus used as a lead compound. GB1101 adjacent to the FSE2 binding site effectively inhibited the expression of PMA-stimulated human TGF-β1 mRNA. PMA stimulates TGF-β1 promoter activity through AP-1 binding sites, not through the FSE2 binding site. The binding of PI polyamides has been reported to alter conformation of the double-strand DNA promoter structure that impairs target promoter activity [14]. PI polyamides suppress the enhanced expression of target transcripts in disease states by the blocking of transcription factor binding and hence the preservation of baseline expression. This is an advantage of PI polyamides because as gene-silencers, side effects are reduced in comparison to nucleic acid medicines such as siRNA and antisense oligonucleotides, which knock down the target gene. We have demonstrated that PI polyamides targeting TGF-β1 promoter effectively ameliorate TGF-β1-induced diseases such as progressive renal diseases [9], restenosis of arteries after injury [10], and encapsulating peritoneal sclerosis [15]. However, the PI polyamides have not yet been developed into practical medicines for clinical use. Thus, we firstly examined the effects of GB1101 on hypertrophic scars in a common marmoset model as a preclinical study, in which efficacy is easily evaluated by visual appearance.

Medicines for hypertrophic scars that are currently in clinical use include tranilast, which is known to inhibit TGF-β1 [16], and corticosteroids [17]. However, tranilast is not specific for TGF-β, and these medicines do not completely improve hypertrophic scars and keloids. Recently, low-molecular weight inhibitors of TGF-β1 receptors, which may be potential practical medicines for TGF-β1-related fibrotic diseases including hypertrophic scars of the skin, have been developed as reagents to define biological effects of TGF-β1. Reports showing that as an inhibitor of TGF-β type I receptor kinase activity, SB-431542 inhibits TGF-β-induced contraction of collagen gels by normal and keloid fibroblasts [18,19], thus suggesting that this compound may have therapeutic potential for treating excessive skin contraction that occurs in keloids. In addition, a novel truncated TGF-β receptor II was shown to possess excellent inhibitory effects on the growth of keloid fibroblasts, their synthesis of collagen type I and TGF-β expression [20]. The same authors showed that treatment with truncated receptor II inhibited TGF-β1 expression both at the mRNA and the protein levels. Nucleic acid medicines that include siRNA are also novel medicines to suppress molecules responsible for various diseases that cannot be rescued with present medicines. Recently, Zhao et al. [21] reported that siRNA-TGF-β1–337 effectively reduced scar fibrosis of the skin and suggested siRNA-TGF-β1–337 as a potential treatment for hypertrophic scars. However, because of its RNA structure, siRNA has the disadvantage of being easily degraded by nucleases *in vivo*.

In the present study, FITC-labeled GB1101 mixed with solbase ointment base was effectively distributed into the skin surface and bound to the nuclei of keratinocytes in the epidermis in rats and marmosets. We repeatedly examined effects of different doses of GB1101 with solbase several times on hypertrophic scarring in 5 marmosets and found a minimum dose of GB1101 with solbase was 30 μg to inhibit the scar formation. In our marmoset model, the 30 μg of GB1101 with solbase completely suppressed hypertrophic scarring at 42 days post-incision accompanied with a reduction in epidermal thickness and in the number of fibroblasts. These findings suggest that the distribution of GB1101 with solbase ointment into injured epidermis is adequate to suppress hypertrophic scars. In comparison to normal skin, vessel formations are enhanced in the dermis in hypertrophic scars [22], which may attribute to the effectiveness of the application of GB1101 with solbase ointment on hypertrophic scars. In the present study, just one application of 30 μg of PI polyamide with solbase ointment completely suppressed the development of hypertrophic scars for 42 days, indicating that quite a small (and cost effective) amount of the PI polyamide targeting to hTGF-β1 is sufficient for ameliorating hypertrophic scars.

The application of PI polyamide to hTGF-β1 with solbase ointment is considered for hypertrophic scars after surgical operations for thyroid diseases, open chest and Caesarean section, and skin burns. Hypertrophic and keloid scars after surgical operations and skin burns often cause psychosocial stresses that can be associated with perceived aesthetic defects [23]. PI polyamide targeting to hTGF-β1 with solbase ointment may improve satisfaction among these patients.

However, PI polyamide targeting to TGF-β1 did not show sufficient inhibition for the treatment of established hypertrophic scars [12]. We injected PI polyamide into rat TGF-β1 into hypertrophic scars at 42 days after incision. As a result, there was a reduction in grossly visible scar elevations, but this effect was not complete. Treatment strongly suppressed TGF-β1 but, according to our histological examination, it did not result in a strong inhibitory effect on fibrosis. It is thought that the transcriptional inhibition of TGF-β1 expression can be inhibited if the PI polyamide is administered before or at the time of incision, at which time it increases promoter activity with AP-1. TGF-β1 promoter activity declines in established scars. Thus, the application of PI polyamide to hTGF-β1 with solbase ointment is considered to be an effective preventive medicine for hypertrophic scars after surgical incision.

Based on results of the present preclinical study, future issues of development of PI polyamides to hTGF-β1 as practical medicines include the development of a large-scale synthesis technique, the evaluation of oncogenesis and hypersensitivity of skin to PI polyamides, and the evaluation of the duration of PI polyamide effectiveness. These points should be confirmed in the development of PI polyamide ointment for hypertrophic scars. Another point to be considered is the aggregation of PI polyamides. Analysis of dynamic light scattering and fractional solubility has shown that PI polyamides have a propensity for aggregation of these compounds [24]. These aggregation properties might induce adverse effects and unexpected pharmacokinetics. This aggregation, however, is seen with high concentrations of PI polyamides and not at low concentrations like those used in treating hypertrophic scars.

In summary, we examined GB1101 as a PI polyamide targeting hTGF-β1 for hypertrophic scars in marmosets. GB1101 with solbase completely suppressed post-incision hypertrophic scarring in comparison to marmosets that received water with solbase. Application of GB1101 with solbase considerably inhibited epidermal thickness and the number of vimentin-positive fibroblasts. Thus, PI polyamide targeting the hTGF-β1 promoter with solbase ointment is suggested to be a practical medicine for patients with hypertrophic scarring after surgical operations and skin burns.

## Supporting Information

S1 Fig Sequences of human and marmoset transforming growth factor (TGF)-β1 promoter analyzed by NCBI BLAST Two-Sequence Analysis. Binding sites (box) of GB1101, GB1105 and GB1106 on the human and marmoset TGF-β1 promoter.
(PDF)

S2 Fig Effects of pyrrole-imidazole (PI) polyamides (GB1101 and GB1106) targeting the human transforming growth factor (TGF)-β1 promoter and mismatch polyamide on expression of TGF-β1 mRNA in marmoset fibroblasts. Marmoset fibroblasts were incubated with 10^-9^ and 10^-7^ M PI polyamides (PIP) targeting human TGF-β1 in the presence or absence of 10^-6^ M phorbol 12-myristate 13-acetate (PMA). Total RNA was extracted, and the expression of TGF-β1mRNAs was evaluated by real time polymerase chain reaction analysis. Data are mean ± SEM (n = 4). * *p* < 0.05 vs. PMA without PI polyamide. # *p* < 0.05 vs. without PMA.
(PDF)
