# Supplementary material for: Preclinical Study of Novel Gene Silencer Pyrrole-Imidazole Polyamide Targeting Human TGF-β1 Promoter for Hypertrophic Scars in a Common Marmoset Primate Model
Source: PLoS One. 2015 May 4;10(5):e0125295. doi: 10.1371/journal.pone.0125295 (PMC4418757; doi:10.1371/journal.pone.0125295)
Supplement: S1 Fig — Binding sites (box) of GB1101, GB1105 and GB1106 on the human and marmoset TGF-β1 promoter. (PDF) [file pone.0125295.s001.pdf]

S1 Figure

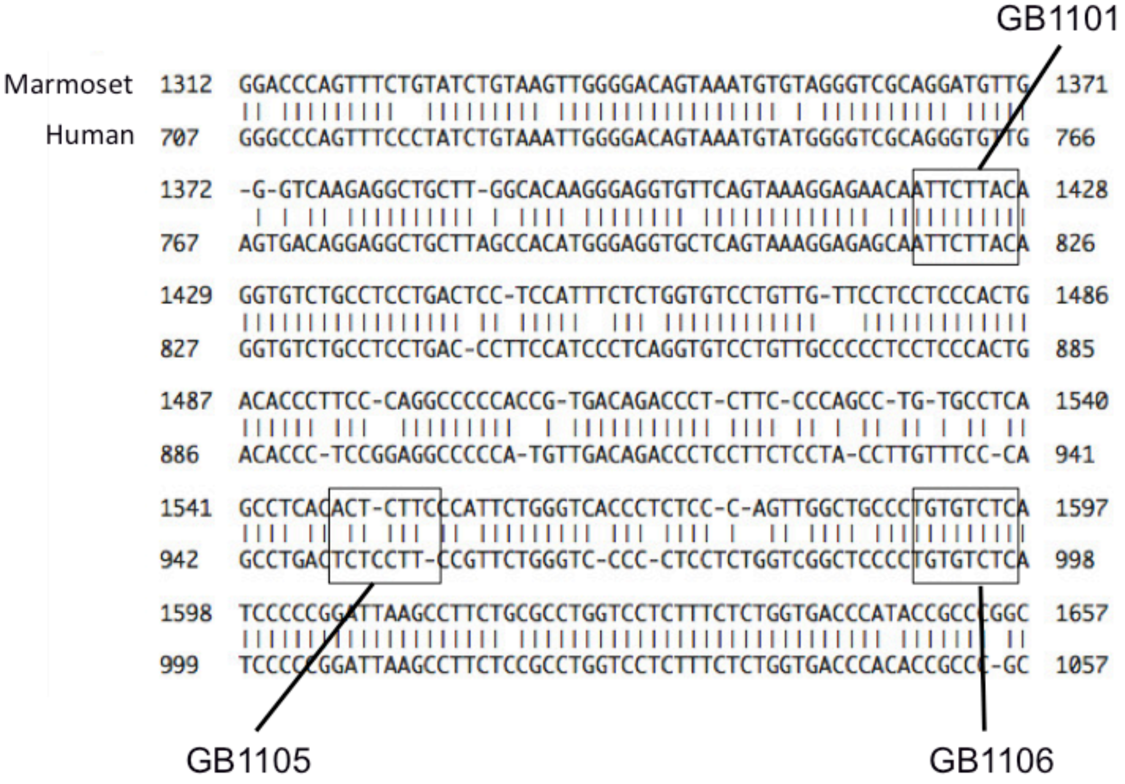

Sequences of human and marmoset transforming growth factor (TGF)-β1 promoter analyzed by NCBI BLAST Two-Sequence Analysis. Binding sites (box) of GB1101, GB1105 and GB1106 on the human and marmoset TGF-β1 promoter.
