# Supplementary material for: Preclinical Study of Novel Gene Silencer Pyrrole-Imidazole Polyamide Targeting Human TGF-β1 Promoter for Hypertrophic Scars in a Common Marmoset Primate Model
Source: PLoS One. 2015 May 4;10(5):e0125295. doi: 10.1371/journal.pone.0125295 (PMC4418757; doi:10.1371/journal.pone.0125295)
Supplement: S2 Fig — Marmoset fibroblasts were incubated with 10-9 and 10-7 M PI polyamides (PIP) targeting human TGF-β1 in the presence or absence of 10-6 M phorbol 12-myristate 13-acetate (PMA). Total RNA was extracted, and the expression of TGF-β1mRNAs was evaluated by real time polymerase chain reaction analysis. Data are mean ± SEM (n = 4). * p < 0.05 vs. PMA without PI polyamide. # p < 0.05 vs. without PMA. (PDF) [file pone.0125295.s002.pdf]

S2 Figure

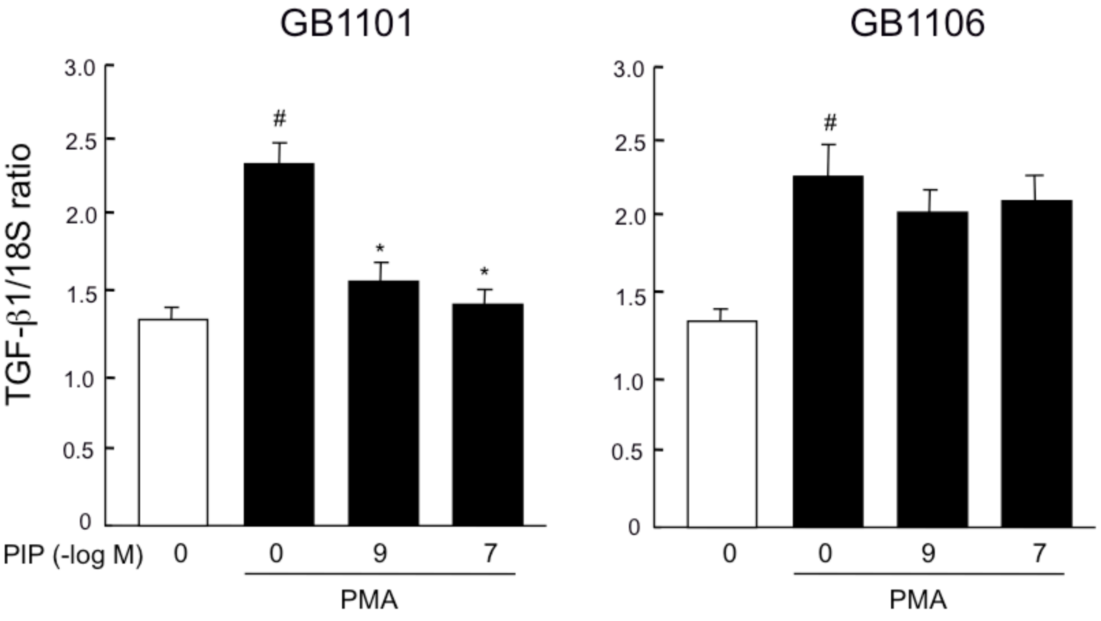

Effects of pyrrole-imidazole (PI) polyamides (GB1101 and GB1106) targeting the human transforming growth factor (TGF)-β1 promoter on expression of TGF-β1 mRNA in marmoset fibroblasts. Marmoset fibroblasts were incubated with 10^{-9} and 10^{-7} M PI polyamides (PIP) targeting human TGF-β1 in the presence or absence of 10^{-6} M phorbol 12-myristate 13-acetate (PMA). Total RNA was extracted, and the expression of TGF-β1mRNAs was evaluated by real time polymerase chain reaction analysis. Data are mean ± SEM (n = 4). * $p < 0.05$ vs. PMA without PI polyamide. # $p < 0.05$ vs. without PMA.
